# Mechanisms on the morphology variation of hematite crystals by Al substitution: The modification of Fe and O reticular densities

**DOI:** 10.1038/srep35960

**Published:** 2016-10-27

**Authors:** Wei Li, Xiaoliang Liang, Pengfei An, Xionghan Feng, Wenfeng Tan, Guohong Qiu, Hui Yin, Fan Liu

**Affiliations:** 1 Key Laboratory of Arable Land Conservation (Middle and Lower Reaches of Yangtse River) Ministry of Agriculture, College of Resources and Environment, Huazhong Agricultural University, Wuhan 430070, PR China; 2 CAS Key Laboratory of Mineralogy and Metallogeny, Guangzhou Institute of Geochemistry, Chinese Academy of Sciences, Guangzhou 510640, PR China; 3 Beijing Synchrotron Radiation Facility, Institute of High Energy Physics, Chinese Academy of Sciences, Beijing 100049, China

## Abstract

Al substitution in hematite is ubiquitous in soils. With the increase of Al amount, the hematite morphology changes from rhombohedral crystals to disk-shaped ones, but the underlying mechanism is poorly understood. Herein, a series of Al-substituted hematite were synthesized and characterized by synchrotron X-ray diffraction (SXRD), field emission scanning electron microscopy (FESEM), high resolution electron transmission microscopy (HRTEM) and extended X-ray absorption fine structure (EXAFS) spectroscopy, to investigate the effects of Al^3+^ substitution on the hematite structure and morphology. EXAFS and Rietveld structural refinement analyses find an increase in face-sharing (along *c* axis) Fe-Me (Me = Al, Fe) distances, edge-sharing (in *a*-*b* plane) Fe-Me (Me = Al, Fe) distances, and O-O average distances. Moreover, the face-sharing Fe-Me distances and O-O distances along *c* axis increase more significantly. This indicates a more apparent decrease in the reticular densities of Fe and O along the direction of *c* axis, which facilitates faster crystal growth along *c* axis and results in the evolution of morphology of Al-substituted hematite to disk-shaped crystals. The above results provide new insights into the morphology changes and environmental geochemistry behaviors of Al-contained hematite in soils, and are benefit for the control of crystal morphologies during its application as environmentally-friendly materials.

As one of the most ubiquitous metal oxides in tropic and sub-tropic soils, hematite has significant effects on the physicochemical properties of soils, due to its various morphologies, particle size, large specific surface area, and high affinity for ions and molecules. Therefore, hematite plays an important role in the geochemical behavior and fate of various nutrients, heavy metals and organic pollutants^1^.

Besides Fe, other metal cations widely exist during the formation and transformation of iron oxides in soils. They can partly replace Fe^3+^ in FeO_6_ octahedron without obviously modifying the crystal structure of iron oxides, owing to their ionic radii and properties similar to those of Fe. Previous studies have shown that various metal cations, e.g., Al^3+^, Ni^2+^, Cu^2+^, Zn^2+^, Cd^2+^, Co^2+^, Mn^2+^, Cr^3+^, Ti^2+^, U^6+^, and Tc^4+^ can be incorporated into the hematite lattice^2^,^3^,^4^,^5^,^6^,^7^,^8^. Among them, Al substitution is the most common in both synthetic and natural hematites.

Aluminum substitution shows great effect on the physicochemical properties of hematite including cell parameters, crystal size, morphology and surface hydroxyl amount, which further modify the surface electrical charge, adsorption of foreign ions, magnetic characters, thermal stability, mineral phase transformation, and soil particle aggregation^1^,^9^,^10^,^11^,^12^,^13^,^14^,^15^,^16^. Especially, the effects of Al substitution on the morphology and specific surface area (SSA) of hematite have drawn wide attention, because they can modify the amount and distribution of surface hydroxyl groups, leading to the alteration of adsorption behaviors towards pollutants, such as phosphorus and znic^17^,^18^. The Al substituted hematites synthesized by co-precipitate method showed thinner disk-shaped crystals with larger diameters with higher Al content, indicating the inhibition effect of Al substitution on the crystal growth of hematite in *c* axis^9^,^17^. The plate-like hematite from the inceptisol, ustiaol and oxisol in Brazil showed a diameter twice the thickness^19^. For synthetic Al substituted hematite and the natural hematite from tropic and sub-tropic soil, the decreases of the ratio of MCD (mean crystalline dimension) (104)/MCD (110) indicates the decreases in the thickness of crystals, with increasing Al substitution^20^,^21^. In the laterite of South China, hematite with 7.7–11.3% Al substitution has a diameter/thickness ratio of 1.2–2.9, while in the red soil and brown red soil, hematite with 8.4–10.3% Al substitution has a ratio of 1.1–1.6, also indicating the thinner hematite with higher Al content^22^. Although the above-mentioned hematite is formed in the various conditions, Al substitution is probably the main cause of plate-like hematite in soils^23^.

The mechanism of metal substitution affecting the morphology of hematite has been discussed previously. Cornell and Giovanoli indicated that, in Cu substituted hematite, the transformation of hematite morphology was caused by the Jahn-Teller effect of Cu^2+^ 24. Without 3d electron, the crystalline field theories cannot be used to explain the effects of Al substitution on the morphology of hematite. In spite of the same valence state and isostructural oxides between Al^3+^ and Fe^3+^, Al substitution results in the distortion of FeO_6_ octahedron and the increase of OH contents in the mineral structure, due to their different ionic radii, which causes lattice strain^23^,^25^. Based on theoretical calculations, the relative stability of crystal faces with low Miller indices can be easily affected by ion adsorption and reaction condition change, because of the similar surface energy of every crystal face1^26^,^27^,^28^,^29^. This suggests that the mechanism of Al^3+^ affecting on the morphology of hematite can be not only explained by lattice strain or anisotropic growth of different crystal faces induced by the adsorption of ion, but also probably connected to the position and properties of substituting metal cations (e.g., radius, electric charge, electronegativity). Additionally, the change of lattice strain and surface energy can be ascribed to the change of reticular density. However, to the best of our knowledge, no study has been reported on the relationship among the Al substitution, reticular density and morphology of hematite.

In this research, a series of Al substituted hematites were characterized by Synchrotron X-ray diffraction (SXRD), Rietveld refinement, field emission scanning electron microscope (FESEM), high resolution electron transmission microscopy (HRTEM) and extended X-ray adsorption fine structure spectroscopy (EXAFS). The effect of the reticular density on the crystal growth were investigated by examining the changes in bond lengths of Fe-Me and O-O pairs after Al incorporation. The results can facilitate a better understanding of the formation of Al-containing hematite in soils and its behavior towards environmentally relevant substances.

## Method and Materials

### Al-substituted hematite synthesis

A certain amount of Fe(NO_3_)_3_·9H_2_O and Al(NO_3_)_3_·9H_2_O (Al/(Al + Fe) = 0.01, 0.03, 0.05, 0.07, 0.09, 0.11, 0.13) were dissolved in 500 mL of deionized water at 90 °C, followed by the addition of KOH solution (1 mol L^−1^, 300 mL) heated at 90 °C and NaHCO_3_ solution (1 mol L^−1^, 50 mL). The mixture was stored in an oven at 90 °C for 96 h. Then, a certain amount of ammonium oxalate (pH = 3.2) was added to the mixture, to remove the amorphous components and impurities. The obtained samples were washed several times with deionized water and dried at 60 °C. Finally, the samples were ground carefully in an agate mortar, passed through a 100 mesh screen, and reserved in polyethylene plastic tubes at room temperature. The obtained samples were named as Hem, AlH1, AlH3, AlH5, AlH7, AlH9, AlH11, and AlH13, respectively.

### Characterization

Synchrotron XRD (SXRD) was performed between 2° and 52° (2*θ*) at BL14B1 of the Shanghai Synchrotron Radiation Facility (SSRF) at a wavelength of 0.6884 Å. A Bede scintillation point detector was employed for data collection in flat plate mode. The step size used was 0.01°. BL14B1 is a bending magnet beamline and uses a Si(111) double-crystal monochromator for energy selection. The typical focused beam size is ~0.2 mm × 0.3 mm. The specific surface area (SSA) was measured with an Autosorb-1 standard physical adsorption analyzer (Quantachrome Autosorb-1). The samples were degassed under vacuum at 110 °C for 3 h to remove water and adsorbate before the adsorption test. The multi-point BET method was used to calculate the SSA. The crystallite morphology of samples was observed by a JSM-6700F field emission scanning electron microscope (FESEM) at an acceleration voltage of 5 kV and an emission current of 5 μA. High resolution electron transmission microscopy (HRTEM) in lattice fringe mode was performed on the samples using a JEM2100F electron microscope operating at 200 kV (resolution in the lattice fringe mode 0.102 nm). For element analysis, an accurate amount of 0.1000 g sample was added into 5 mL of HCl solution (5 mol L^−1^), and heated on an electric stove until the particles were completely dissolved. The component concentration was analyzed using ICP-MS.

### SXRD Rietveld refinement

SXRD Rietveld refinement was performed with Materials studio 8.0. The refine values (cell parameters *a* and *c*, atomic positions, site occupancies, thermal parameters) were used as the starting model with the position of Al incorporating equally to that of Fe. The Pseudo-Voigt function was used to present the individual reflection profiles, allowing us to deconvolute the strain broadening contributions. The preferred orientation was corrected with the March-Dollase function.

### EXAFS

The EXAFS spectra of Al-substituted hematite were measured at room temperature on the 1W1B beamline at the Beijing Synchrotron Radiation Facility (BSRF). Fe K-edge EXAFS data were collected over the energy range of 6953–7884 eV in transmission mode. A Fe metal foil reference was collected (adsorption edge jump at 7112 eV) to calibrate the monochromator before every sample run.

The analysis of all EXAFS data were performed using IFEFFIT/SixPack^30^. For Fe K-edge spectra, averaged spectra were background-subtracted using the following parameters: E_0_ = 7127 eV, R_bkg_ = 0.95, and k-weight = 2. Structural parameters (R, CN, and Debye-Waller factor, σ^2^) were obtained by fitting the experimental K^3^-weighted EXAFS spectra to the standard EXAFS equation^31^. Phase and amplitude functions for single-scattering paths were calculated using FEFF7^32^. The EXAFS fittings were conducted over k range of 3–13 Å and R range of 1–4 Å, with an amplitude reduction factor (S_0_^2^) of 0.80 from Yin *et al*.^33^. In all the fits, the number of independent variables used was less than the number of independent data points. During the Fourier transformation and EXAFS data fitting, a Hanning window was used.

## Results

### Elemental analysis and SSA

The elemental analysis and SSA results of Al-substituted hematite samples are listed in Table 1. Al substitution results in a lower final Al content than the initial one, with the highest value being 9.23% in AlH13. With the increase of Al substitution, the SSA generally shows an upward trend.

### SXRD and Rietveld refinement

**Figure 1:**
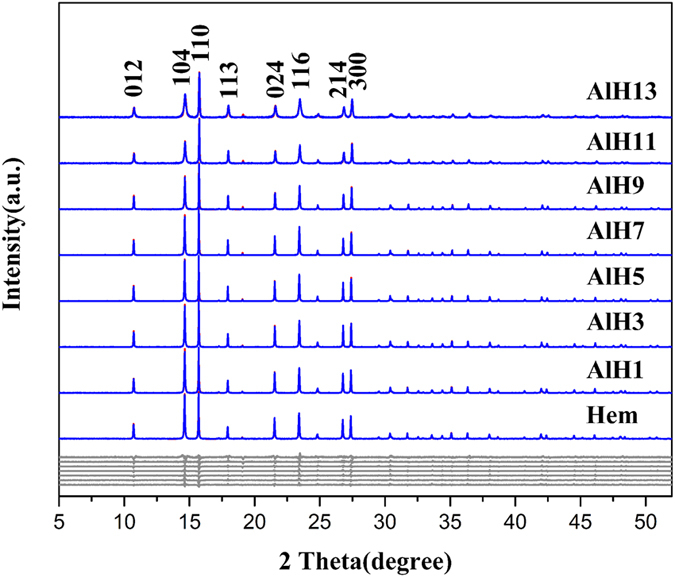
Rietveld structural refinement of Al-substituted hematite samples (Blue lines: experimental data; red lines: calculated patterns; gray lines: difference patterns).

The SXRD patterns of Al-substituted hematite samples are presented in Fig. 1. All the diffraction peaks can be indexed to α-Fe_2_O_3_ (JCPDS 33-0664), suggesting that the prepared samples are pure phase. With Al content increase, the intensity of each peak initially increases and then decreases. The relative intensities of these peaks also change. For example, the ratio of (104)/(110) and (214)/(300) decreases.

The Scherrer formula is used to calculate the MCDs of (104) and (110) (Table 2)^34^. With the Al substitution increase, both MCD (104) and MCD (110) initially increase, but followed by an apparent decrease. The MCD (104) was used to represent the crystal size along *c* axis, while MCD (110) was used to represent the crystal size in *a*-*b* plane. The ratio of MCD (104)/MCD (110) decreases from 0.70 for Hem to 0.46 for AlH13, indicating that the crystal morphology of hematite gradually transforms to plate^17^.

**Figure 2:**
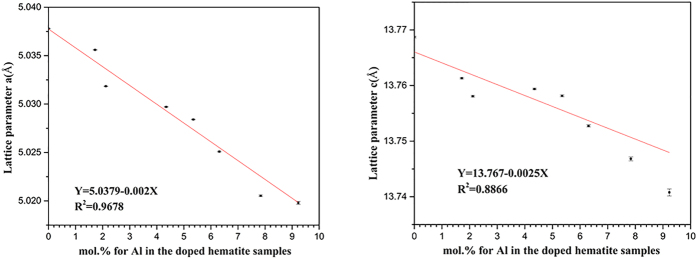
The relationship between Al substitution amount and cell parameters *a* (left) and *c* (right).

The Rietveld structure refinement was conducted based on the hematite crystal model (JCPDS: 33-0664). The results are shown in Figs 1 and 2, and Tables 3 and 4. The unit cell parameters *a* and *c* gradually decrease. For example, *a* decreases from 5.0378 Å for Hem to 5.0199 Å for AlH13, while *c* decreases from 13.7689 Å to 13.7414 Å. For all the Al-doped hematite samples, an excellent linear relationship exists between the Al contents and cell parameters *a* (n = 9, R^2^ = 0.96778 ˃ 0.8343, p < 0.01) or *c* (n = 9, R^2^ = 0.8866 ˃ 0.8343, p < 0.01) (Fig. 2). Vandenberghe *et al*. also discovered the similar linear relationship between the Al substitution extent and cell parameters *a* or *c*, respectively^35^.

**Figure 3:**
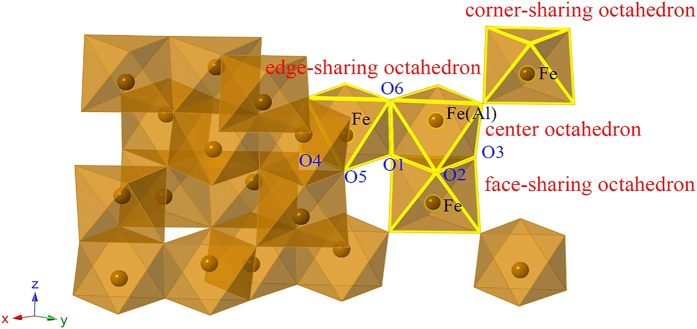
Schematic description of Fe coordination environment in hematite.

The O-O distance in the hematite structure is presented in Table 4. The O1-O2, O1-O3, O2-O3, O1-O4, O1-O5, O4-O5 bonds are parallel to *a*-*b* plane, while O1-O6 bond is parallel to *c* axis (Fig. 3). With increasing Al content, the distances of O1-O2, O1-O3, O2-O3, O1-O6 increase while those of O1-O4, O1-O5, O4-O5 decrease.

### Morphology

**Figure 4:**
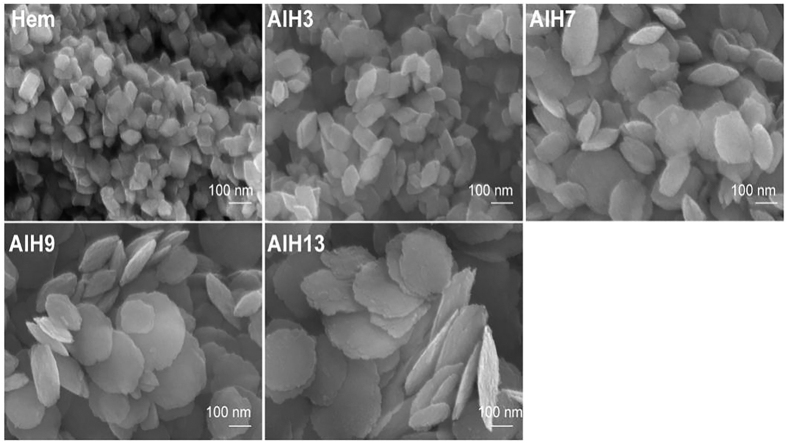
SEM images of hematite and Al-substituted hematite samples.

The SEM images of Al-doped hematite samples are presented in Fig. 4. The morphology of Hem is rhombohedral. For the samples with low Al content (AlH3), the crystal remains rhombohedral. However, with Al amount increase, its morphology transforms from rhombohedral to disk-shaped plate, with a smaller thickness and a larger diameter (AlH9 and AlH13), which is consistent with the XRD results.

**Figure 5:**
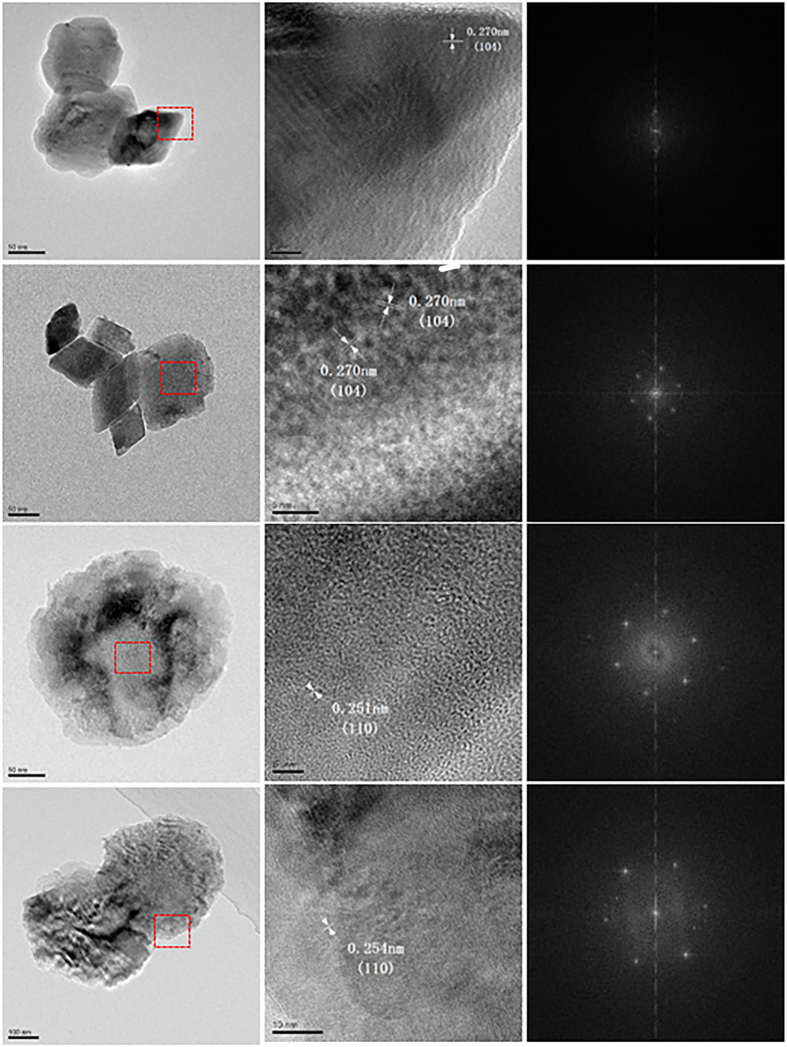
HRTEM images of the samples (from top to bottom: Hem, AlH5, AlH11, AlH13) together with their fast Fourier filtered-selected area electron diffraction.

The HRTEM images of Al-doped hematite are presented in Fig. 5. The distance between two adjacent fringes is calculated. Without Al content or with low Al content, the fringes correspond to the crystallographic plane (104) of hematite. However, with higher Al content, the fringes correspond to the crystallographic plane (110) of hematite, which indicates the decrease of thickness along *c* axis. It should be pointed out that the small variation in the certain atom bindings that is excepted as a function of Al doping cannot be distinguished by HRTEM^36^.

### Fe K-edge EXAFS

**Figure 6:**
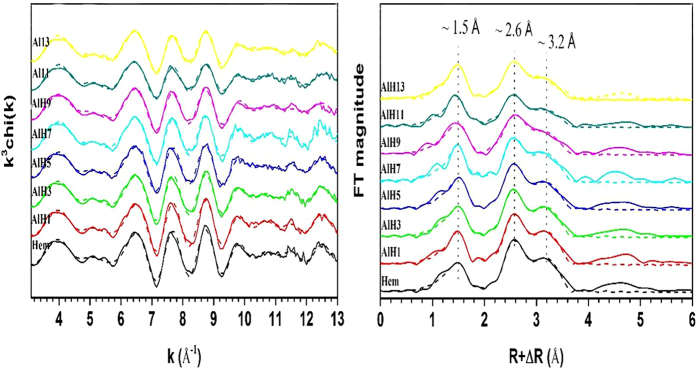
Fe K-edge EXAFS spectra (left) and Fourier transformed spectra (FTs, right) of hematite and Al-substituted hematite samples (the solid lines: experimental data; dash lines: best fits).

The EXAFS and FT spectra of the Al-substituted hematite samples are presented in Fig. 6. For the hematite samples, their spectra are similar, indicating that the Al substitution does not change the basic structure of hematite. The three peaks on the FT spectra are related to the Fe-O shell (R + ΔR~1.5 Å), face-sharing Fe-Fe_F_ and edge-sharing Fe-Fe_E_ shell (R + ΔR~2.6 Å), and corner-sharing Fe-Fe_C_ shell (R + ΔR~3.2 Å), respectively^1^. Considering the low atomic number, the small scatting factor and uncertain position of Al^3+^ in hematite, the attribution from Al^3+^ was not considered during the EXAFS fitting. The best fits are depicted in the dotted lines in Fig. 6, overlaid with the experimental data. The fitting results are summarized in Table 5. With increasing Al content, the Fe-O, Fe-Fe_E_ and Fe-Fe_C_ distances increase slightly, while the Fe-Fe_F_ distance increases apparently.

## Discussion

In this study, with the increase of Al substitution, the cell parameters decrease while the Fe-Fe distances increase. This is similar to the observation in previous work^3^. But so far, the mechanism has not been elucidated.

**Figure 7:**
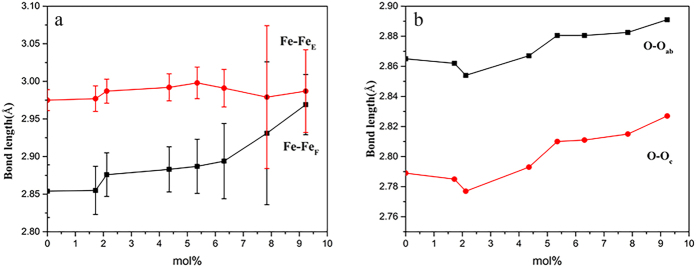
The bond lengths of Fe-Fe(left) and O-O(right) versus Al substitution in hematite and Al-substituted hematite samples.

Because of the smaller ionic radius of Al^3+^ (r = 0.535 Å) than Fe^3+^ (r = 0.645 Å)^37^, the substitution of Al^3+^ for Fe^3+^ results in smaller AlO_6_ octahedron. Moreover, the formation of Al-O-Fe atomic chain causes the increase in electron cloud density of Fe-O, and the decrease in the distances of Fe-O bonds, ascribed to the weaker electronegativity of Al^3+^ (χ = 1.499) than Fe^3+^ (χ = 1.687)^38^. So the Fe atom would be closer to the Al atom, leading to the decrease in the distance of Al-O-Fe bond and the increase in the distances of adjacent Fe-O-Fe (Al-O-Fe-O-Fe) bonds (Figs 3 and 7a). The unit cell of Al-substituted hematite consists of AlO_6_ and FeO_6_ octahedrons. Due to the smaller AlO_6_ octahedrons than FeO_6_ ones, and shorter Al-Fe distances than Fe-Fe, the unit cell parameters of hematite decrease with increasing Al substitution. The decrease in Al-Fe bond distance will cause the increase in several adjacent Fe-Fe bond distances. The Fe-Fe distances obtained from Fe K-edge EXAFS analysis are the average Fe-Fe distances, from which the Al-Fe distances cannot be distinguished. Thus, as revealed by EXAFS fitting, the Fe-Fe distances increase with increasing Al amount.

The strong repulsion force in the Fe-Fe pairs is responsible for the distortion of face-sharing FeO_6_ octahedrons, making the O-O distances along the shared face of an octahedron shorter^1^. The weaker electronegativity of Al^3+^ than Fe^3+^ results in a smaller repulsion force in Al-Fe pairs than in Fe-Fe pairs. Consistent with the result of Rietveld structure refinement, after Al substitution in FeO_6_ octahedron, the O1-O2, O1-O3, O2-O3 distances along the shared face of an octahedron increase (Figs 3 and 7b), making the distances closer to the O1-O6 distance than those in pure hematite (Table 4). This indicates that the Al substitution reduces the distortion of face-sharing Fe(Al)O_6_ octahedron.

EXAFS results show that, with increasing Al content, the Fe-Fe_E_ and Fe-Fe_C_ distances increase slightly, and the Fe-Fe_F_ distance increases obviously (Table 5). Due to the greater repulsion force in the Fe-Fe bond in face-sharing FeO_6_ octahedrons and the instability of face-sharing FeO_6_ octahedrons, the Al substitution in FeO_6_ octahedrons facilitates the adjustment of face-sharing FeO_6_ octahedrons, leading to the remarkable change of face-sharing Fe-Fe distances.

**Figure 8:**
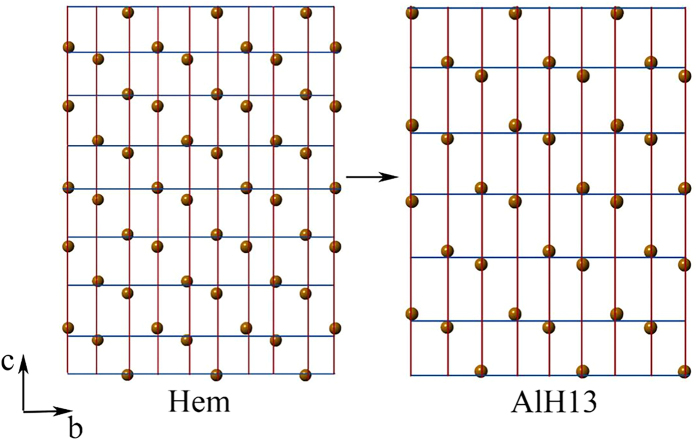
Diagrams of Fe reticular density of b-, c- axis for hematite and Al substituted hematite (AlH13) samples.

As shown by the EXAFS results, with increasing Al substitution, the edge-sharing Fe-Fe distance increases by 0.4%, but the face-sharing Fe-Fe distance increases by 4.0% (Fig. 7a). In the hematite structure, the face-sharing FeO_6_ octahedrons are parallel to *c* axis, and edge-sharing octahedrons are parallel to *a*-*b* plane. This indicates that the Al substitution causes the remarkable decrease of Fe reticular density along the *c* axis, and the slight decrease of Fe reticular density along the *a*-*b* plane (Fig. 8).

The Rietveld structure refinement result (Table 4) show ca. 9.08‰ increase in the O-O_ab_ distance, but ca. 13.63‰ increase in the O-O_c_ distance. This illustrates that Al substitution causes a more significant decrease of O reticular density along *c* axis than along *a*-*b* plane (Fig. 7b), which is in line with the change of Fe reticular density.

According to the “Law of Bravais”, during crystal growth, the difference between reticular densities of atoms causes the changes of attractive force between particles in the medium and the difference in the growth rate of relevant faces. The decrease of the reticular density results in the increase of both the attractive force and growth rate of the relevant faces^39^,^40^,^41^. In the Al substituted hematite, the Fe and O reticular densities along *c* axis (nonbasal faces) decrease more obviously than those along *a*-*b* plane (basal faces) (Fig. 8), leading to a faster growth of the nonbasal faces than the basal faces^17^, and a decrease of the crystal size along *c* axis. Therefore, the crystal becomes thinner.

It is well known that hematite nanostructure synthesized by hydrothermal or hydrolysis methods usually grows by the combination of diverse crystal growth mechanisms, including Ostwald ripening (OR) and oriented attachment (OA) of nanocrystals^42^,^43^. But the connections between Fe, O reticular densities and samples morphology indicate that the effect of OR may play a leading role in the modification of Al-doped hematite morphology.

## Conclusion

In this study, the mechanisms of Al substitution on hematite morphology were explored. Owing to the smaller ionic radius and weaker electronegativity of Al^3+^ than Fe^3+^, the Al substitution adjusts the shape of the face-sharing FeO_6_ octahedrons and changes, to a varying degree, the Fe and O reticular densities of nonbasal and basal faces, which leads to the transformation of hematite crystal from rhombohedral to plate-like. The results presented here provide insights into the underlying interaction mechanisms between metal cations and iron oxide minerals in soils, and also offer useful reference for morphology modification in the design and fabrication of environment-friendly metal oxide adsorbents and catalysts.

## Additional Information

**How to cite this article**: Li, W. *et al*. Mechanisms on the morphology variation of hematite crystals by Al substitution: The modification of Fe and O reticular densities. *Sci. Rep.* **6**, 35960; doi: 10.1038/srep35960 (2016).

**Publisher’s note:** Springer Nature remains neutral with regard to jurisdictional claims in published maps and institutional affiliations.

## Figures and Tables

**Table 1:** The Al content and SSA of Al-substituted hematite samples.

Sample	Hem	AlH1	AlH3	AlH5	AlH7	AlH9	AlH11	AlH13
Al mol%	0	1.72	2.12	4.35	5.35	6.31	7.84	9.23
SSA (m^2^/g)	24	21	25	21	25	24	59	34

**Table 2:** The mean crystallite dimensions (MCDs) of (104) and (110) crystal faces of Al-substituted hematite samples.

Sample	MCD(104)(nm)	MCD(110) (nm)	MCD(104)/MCD(110)
Hem	45.37	64.43	0.70
AlH1	45.37	68.14	0.67
AlH3	49.16	73.83	0.67
AlH5	58.02	84.37	0.69
AlH7	52.82	77.04	0.69
AlH9	48.48	80.54	0.60
AlH11	32.18	72.32	0.44
AlH13	22.83	49.91	0.46

**Table 3:** Unit cell parameters of Al-substituted hematite samples obtained by Rietveld structure refinement analysis.

Sample	Atom	Position			Unit cell Parameters (Å)		
		x	y	z	*a*	*c*	R_wp_ (%)
Hem	Fe	0	0	0.35446 (4)	5.03779 (3)	13.76870 (14)	7.52
	O	0.31236 (47)	0	0.25			
AlH1	Fe	0	0	0.35469 (4)	5.03560 (3)	13.76131 (13)	8.3
	O	0.31157 (40)	0	0.25			
AlH3	Fe	0	0	0.35431 (4)	5.03184 (3)	13.75806 (13)	7.56
	O	0.30854 (36)	0	0.25			
AlH5	Fe	0	0	0.35351 (4)	5.02971 (3)	13.75937 (12)	7.88
	O	0.31557 (36)	0	0.25			
AlH7	Fe	0	0	0.35248 (5)	5.02841 (4)	13.75815 (15)	8.86
	O	0.32247 (39)	0	0.25			
AlH9	Fe	0	0	0.35214 (6)	5.02509 (5)	13.75274 (19)	10.29
	O	0.32344 (44)	0	0.25			
AlH11	Fe	0	0	0.35134 (8)	5.02053 (8)	13.74682 (36)	12.42
	O	0.32567 (52)	0	0.25			
AlH13	Fe	0	0	0.35073 (9)	5.01980 (16)	13.74078 (64)	15.4
	O	0.32944 (60)	0	0.25			

**Table 4:** O-O distances of Al-substituted hematite samples obtained by Rietveld structure refinement analysis.

	O1-O2	O1-O3	O2-O3	O1-O4	O1-O5	O4-O5	O-Oab*	O1-O6	O-Oc*
Hem	2.726 (5)	2.726 (5)	2.726 (5)	3.004 (2)	3.004 (2)	3.004 (2)	2.865	2.789 (2)	2.789 (2)
AlH1	2.717 (3)	2.717 (3)	2.717 (3)	3.007 (2)	3.007 (2)	3.007 (2)	2.862	2.785 (1)	2.785 (1)
AlH3	2.689 (3)	2.689 (3)	2.689 (3)	3.019 (2)	3.019 (2)	3.019 (2)	2.854	2.777 (0)	2.777 (0)
AlH5	2.750 (4)	2.750 (4)	2.750 (4)	2.984 (2)	2.984 (2)	2.984 (2)	2.867	2.793 (1)	2.793 (1)
AlH7	2.809 (4)	2.809 (4)	2.809 (4)	2.952 (1)	2.952 (1)	2.952 (1)	2.8805	2.810 (1)	2.810 (1)
AlH9	2.815 (4)	2.815 (4)	2.815 (4)	2.946 (1)	2.946 (1)	2.946 (1)	2.8805	2.811 (1)	2.811 (1)
AlH11	2.832 (5)	2.832 (5)	2.832 (5)	2.933 (2)	2.933 (2)	2.933 (2)	2.8825	2.815 (1)	2.815 (1)
AlH13	2.870 (5)	2.870 (5)	2.870 (5)	2.912 (2)	2.912 (2)	2.912 (2)	2.891	2.827 (1)	2.827 (1)

^*^O-O_ab_ is the average of O1-O2, O1-O3, O2-O3, O1-O4, O1-O5, O4-O5 bonds, while the O-O_c_ is O1-O6 bond.

**Table 5:** Structure parameters derived from the fitting of Fe K-edge EXAFS spectra of hematite and Al-doped hematite samples.

	Hem	AlH1	AlH3	AlH5	AlH7	AlH9	AlH11	AlH13
Fe-O1 (1)								
CN	3	3	3	3	3	3	3	3
R (Å)	1.933 (15)	1.942 (17)	1.939 (10)	1.939 (11)	1.942 (12)	1.939 (12)	1.937 (11)	1.938 (12)
σ2 (Å)	0.0047 (15)	0.0052 (18)	0.0036 (8)	0.0036 (9)	0.0037 (11)	0.0034 (10)	0.0038 (9)	0.0036 (9)
Fe-O1 (2)								
CN	3	3	3	3	3	3	3	3
R (Å)	2.078 (21)	2.076 (27)	2.090 (17)	2.088 (19)	2.089 (22)	2.089 (21)	2.087 (18)	2.093 (21)
σ2 (Å)	0.0064 (24)	0.0087 (43)	0.0066 (18)	0.0069 (9)	0.0072 (25)	0.0067 (22)	0.0063 (17)	0.0068 (19)
Fe-FeF								
CN	1	1	1	1	1	1	1	1
R (Å)	2.854 (35)	2.855 (32)	2.876 (29)	2.883 (30)	2.887 (36)	2.894 (50)	2.931 (95)	2.969 (40)
σ2 (Å)	0.0019 (10)	0.0016 (24)	0.0024 (15)	0.0023 (27)	0.00323 (31)	0.0033 (50)	0.0078 (62)	0.0051 (40)
Fe-FeE								
CN	3	3	3	3	3	3	3	3
R (Å)	2.975 (14)	2.977 (17)	2.987 (16)	2.992 (18)	2.998 (21)	2.991 (25)	2.979 (95)	2.987 (55)
σ2 (Å)	0.0019 (10)	0.0025 (13)	0.0035 (13)	0.0035 (14)	0.0034 (16)	0.0041 (23)	0.0063 (17)	0.0118 (46)
Fe-FeC (1)								
CN	3	3	3	3	3	3	3	3
R (Å)	3.391 (11)	3.397 (13)	3.397 (10)	3.399 (11)	3.403 (13)	3.398 (13)	3.398 (13)	3.410 (17)
σ2 (Å)	0.0038 (8)	0.0044 (9)	0.0050 (7)	0.0051 (8)	0.0052 (9)	0.0052 (9)	0.0069 (11)	0.0067 (12)
Fe-FeC (2)								
CN	6	6	6	6	6	6	6	6
R (Å)	3.687 (13)	3.683 (14)	3.685 (12)	3.688 (14)	3.693 (16)	3.686 (16)	3.688 (17)	3.679 (22)
σ2 (Å)	0.0086 (9)	0.0092 (11)	0.0105 (10)	0.0111 (11)	0.0114 (13)	0.0112 (13)	0.0133 (16)	0.0147 (23)
E0 (eV)	−2 (2)	−1 (2)	0 (2)	1 (2)	1 (2)	1 (2)	0 (2)	1 (2)
Chi Sq	44	72	797	1118	128	63	22	21
R-factor	0.0048	0.0045	0.0033	0.0038	0.0049	0.0048	0.0048	0.008
